# Association between gubernacular canals characteristics and teeth eruption status: a cone-beam computed tomography study

**DOI:** 10.4317/jced.61169

**Published:** 2024-02-01

**Authors:** Emad Behrouzi, Farida Abesi, Hakimeh Ghorbani, Hemmat Gholinia

**Affiliations:** 1 Student Research Committee, Babol University of Medical Sciences, Babol, Iran; 2 Dental Materials Research Center, Oral and Maxillofacial Radiology Department, Faculty of Dentistry, Babol University of Medical Sciences, Babol, Iran; 3 Oral Health Research Center, Health Research Institute, Babol University of Medical Sciences, Babol, Iran; 4 Health Research Institute, Babol University of Medical Sciences, Babol, Iran

## Abstract

**Background:**

There have been few studies that have evaluated the imaging characteristics of the gubernacular canal. Additionally, it is important to understand the role of this structure and its relationship with tooth erupt. Therefore, the objective of this study was to investigate the association between gubernacular canal features and teeth eruption status on cone-beam computed tomography (CBCT) images.

**Material and Methods:**

In this cross-sectional study, 150 CBCT images were obtained from patients referred to a maxillofacial radiology clinic in Babol, northern Iran, in 2021. Eruption status (normal, delayed, and impacted) and the presence of the gubernacular canal were recorded. If the gubernacular canal was detected, its opening site in the alveolar crest (buccal, lingual, and central) and its attachment site to the dental follicle (usual, unusual) were further assessed.

**Results:**

Gubernacular canal was observed in 133 (88.7%) of the total 150 CBCT images. Also, 41 cases (27.3%) had impacted teeth. The detection rate of the gubernacular canal in the normal, delayed, and impacted eruption of teeth was 92.1% (n=93), 75.0% (n=6), and 82.9% (n=34), respectively (*p*=0.135). Opening the gubernacular canal in the alveolar crest was not associated with teeth eruption status. Also, unusual attachment sites of the gubernacular canal to dental follicles were mostly seen in abnormal eruptions.

**Conclusions:**

According to the findings, observing the presence of the gubernacular canal on CBCT may not help anticipate teeth eruption problems.

** Key words:**Gubernacular canal, Cone-beam computed tomography, Tooth eruption.

## Introduction

The gubernacular canal or gubernacular tract is an alveolar bony canal that contains the gubernacular cord, a fibrous connective tissue containing peripheral nerves, blood, lymph vessels, and epithelial cells of the dental lamina. The canal, which connects the permanent tooth’s pericoronal follicular tissue (dental sac) to the alveolar crest and the overlying gum, is located relative to the palatal of the deciduous tooth (1,2). The gubernacular canal has been stated to have a potential effect on the normal growth of permanent teeth (3,4). However, there are controversies on the role of the gubernacular canal in induction of normal eruption (5,6). Gubernacular cord remnants can also be the basis for developing some odontogenic cysts and tumors. Pathological and imaging studies have provided robust data proposing that the gubernacular canal may be the source of adenomatoid odontogenic tumors and odontoma (7,8).

Cone-beam computed tomography (CBCT) system is an imaging technique that provides three-dimensional images with high resolution, allowing clinicians to have 360-degree views of the oral and maxillofacial anatomy with high accuracy (9,10). In CBCT images, the gubernacular cord is observed in the axial view as low-density round areas on the lingual side. The diameter of these areas is about 1 mm. In the coronal and sagittal view, it is seen as a low-density cortical area attached to an undeveloped tooth follicle (11). The more teeth erupt, the smaller the gubernacular cord is seen (12).

Because of their high-risk radiation, CBCT images are not routinely used in clinical practice, especially for children. However, dental practitioners consider CBCT as a precious method for diagnosing and treatment planning in some instances in orthodontics, such as interceptive orthodontics or surgical treatments for teeth with positional or eruption anomalies (13,14). Volumetric data are beneficial in identifying the morphology of the tooth and its relationship with its adjacent structures, thus contributing to clinical decision-making (for example, in the case of extraction or extrusion of the tooth) (15). Although CBCT is not routinely allowed to check for the presence of the gubernacular canal, it has been used for other reasons, such as orthodontics (6).

A limited number of studies have investigated the imaging characteristics of the gubernacular canal. Also, it is needed to elucidate the role of this structure and its relationship with tooth eruption. Thus, in the present study, we aimed to investigate the link between gubernacular canal features and teeth eruption status on CBCT images.

## Material and Methods

-Participants, locations, and imaging

**Figure 1:**
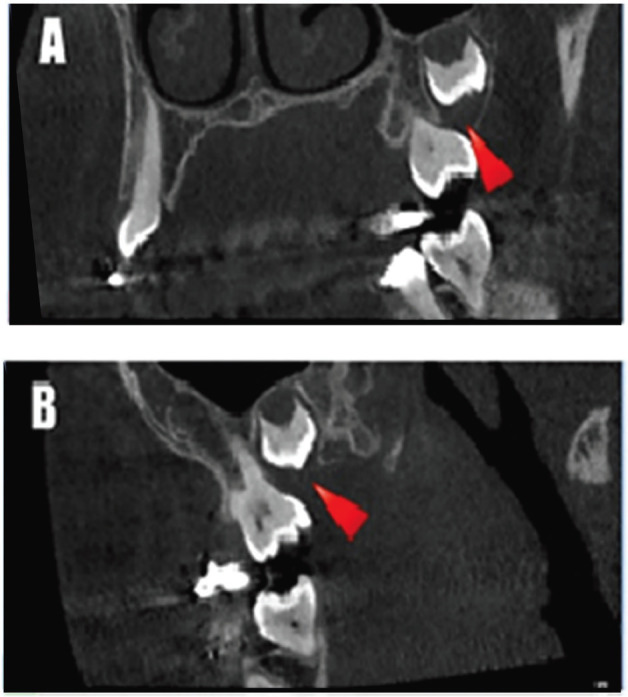
Gubernacular canal detected in third upper molar tooth in coronal (A) and sagittal (B) views.

In this cross-sectional study, 150 CBCT images were obtained from patients referred to a maxillofacial radiology clinic in Babol, northern Iran, in 2021. We assessed the molar, premolar, and canine teeth. The scans were evaluated for the characteristics of the gubernacular canals. We did not apply an age range limitation. We excluded the scans displaying movement artifacts, supernumerary teeth (since these teeth do not have a defined eruption pattern), and teeth in an advanced eruption stage (i.e., cusps beyond the level of the alveolar crest). We prepared the CBCT images using Giano Newtom (Verona, Italy) with specific parameters, including 90 KvP, 10 mAs, focal spot of 0.05 mm, scan time of 18-26 seconds, x-ray emission time of 3.6 seconds, axial thickness of 0.300 mm, and a field of view measuring 11 x 13 cm. Images were taken in 0.5 mm sections with intervals of 1 mm. In sagittal and coronal views, the presence of gubernacular canals, their size and diameter, and their connection to the tooth in the bone were determined and recorded (Fig. 1).

-Sample size calculation

**Figure 2:**
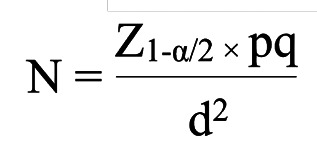
Formula

The sample size was estimated as 150 subjects using the following formula, where α=0.05, *p*=0.6, q=0.4 and d=0.08, (Fig. 2):

-Data collection

**Figure 3:**
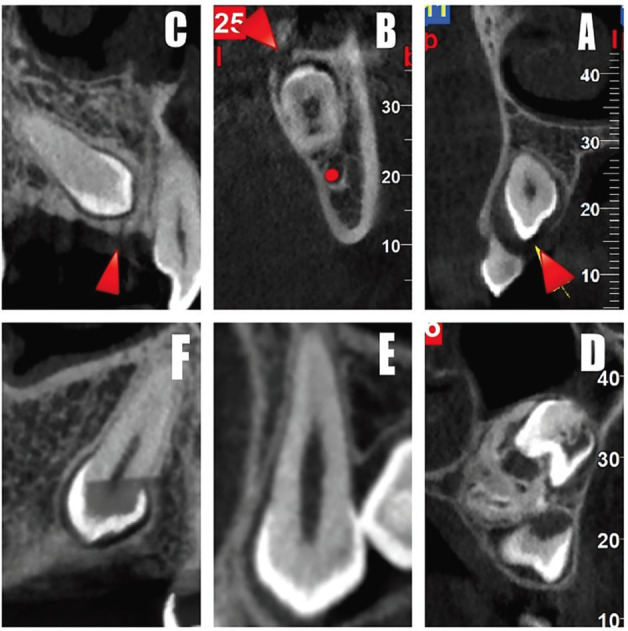
Cone-beam computed tomography images of the gubernacular canal were detected in teeth with normal eruption (A), delayed eruption (B) and impacted tooth (C), and not detected in normal eruption (D), delayed eruption (E) and impacted tooth (F).

In this study, patient sex and age, eruption status (normal, delayed, and impacted), the presence of the gubernacular canal, and its length and diameter were recorded. If the gubernacular canal was detected, its opening site in the alveolar crest (buccal, lingual, and central) and attachment site to the dental follicle (usual, unusual) were further assessed. In the buccal-lingual direction, there are three attachment sites of gubernacular canal to the dental follicle, including buccal, lingual, and central. Regarding eruption status, teeth are classified as impacted if a physical barrier is detected (such as lack of space in the dental arch, or deviated dental germ). Delayed eruption refers to the condition in which the tooth is intraosseous, without any visible physical barriers, and also the difference between the patient’s age and the mean eruption age of the dental group in the study population is more than twice the standard deviation established for that dental group (16,17). Figure 3 shows the CBCT images of gubernacular canals detected in teeth with different eruption statuses. With respect to recording the length and diameter of the gubernacular canal, we considered the length of the canal between the perpendicular line from the branching point of the canal from the tooth to the line connecting the closest parts of the alveolar crest to the surface on both sides of the canal; the diameter of the canal was recorded as the largest size observed in the CBCT image. The mean eruption ages that served as references in our study were obtained from valid references (18). Teeth that had no visible physical barriers and the patient’s age was within the mean eruption age were considered a normal eruption (6).

-Statistical Analysis

The collected data underwent statistical analyses using SPSS v22. The descriptive analysis was used to calculate frequency, percentage, mean, and standard deviation. We also used the chi-squared test to compare the categorical variables. The ANOVA was utilized to compare the means between the different groups. A *p-value* less than 0.05 was considered to be significant.

-Ethical issues

We obtained a written consent form from all participants. The present research was approved by the Research Ethics Committee of Babol University of Medical Sciences (ethics code: IR.MUBABOL.HRI.REC.1400.105).

## Results

Of 150 cases, 81 (54%) were women, and 69 (46%) were men. The mean age of the patients was 18.57±11.27 years, ranging from 6 to 55 years. Forty-two subjects (28%) were aged ≤14 years and 108 (72%) were older than 14 years. Table 1 summarizes the gubernacular canal distribution by teeth eruption status. As represented, the gubernacular canal was observed in 133 (88.7%) of the total 150 CBCT images. Also, 41 cases (27.3%) had impacted teeth. The detection rate of the gubernacular canal in the normal, delayed, and impacted eruption of teeth was 92.1% (n=93), 75.0% (n=6), and 82.9% (n=34), respectively.

**Table 1:**
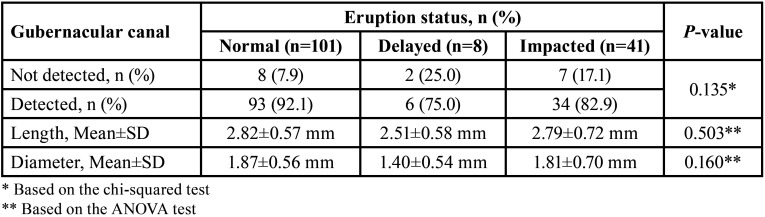
Distribution, length, and diameter of gubernacular canal by teeth eruption status.

The mean length and diameter of the gubernacular canals were 2.80±0.61 mm (ranging from 1.50 to 4.90 mm) and 1.84±0.60 mm (ranging from 0.40 to 3.40 mm). The results of measuring the length and diameter of the gubernacular canal according to the eruption status are presented in Table 1. The length and diameter of the gubernacular canals differed between the normal, delayed, and impacted eruption of teeth, but no significant differences were found.

Table 2 summarizes the distribution of gubernacular canals opening in the alveolar crest seen in teeth with (anterior/premolars) and without (molars) predecessor primary tooth according to the teeth eruption status. The anterior and premolar groups were pooled since they represented teeth with primary predecessors. In anterior/premolars, there were 15 cases with impacted teeth, of whom 12 cases had lingual, one had central, and two had a buccal opening. In molar teeth, there were 19 cases with impacted teeth, of which one case showed a lingual opening, 17 were related to a central opening, and one pertained to a buccal opening.

**Table 2:**
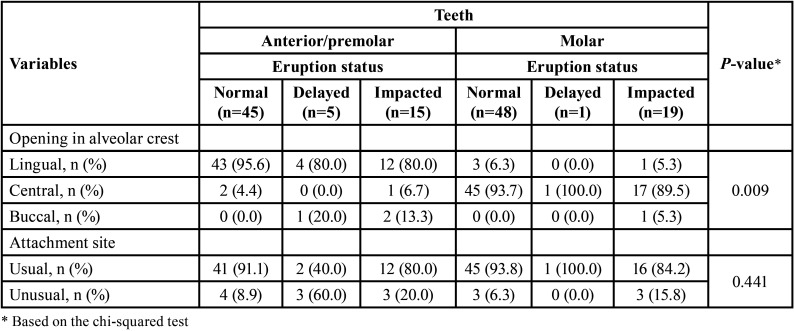
Distribution of gubernacular canal opening in alveolar crest and attachment site to dental follicle seen in teeth with (anterior/premolars) and without (molars) predecessor primary tooth according to teeth eruption status.

**Figure 4:**
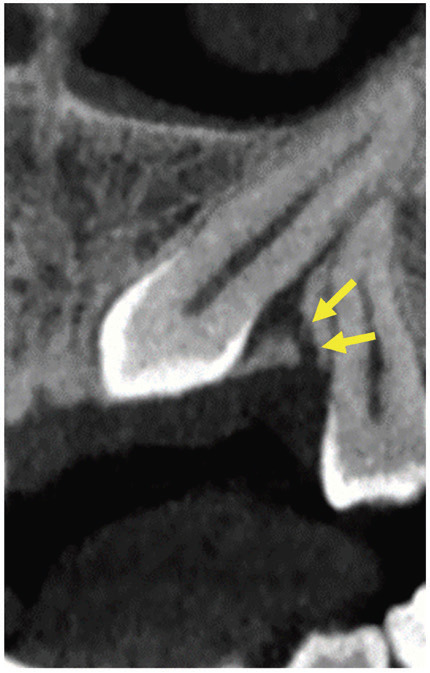
An example of an unusual attachment site of the gubernacular canal to the dental follicle in the upper canine. The canal is attached in lingual (buccal-lingual aspect) and central (occlusal-cervical aspect) direction to the dental follicle.

Table 2 also represented the distribution of gubernacular canals attachment site to dental follicle seen in teeth with (anterior/premolars) and without (molars) predecessor primary tooth according to the teeth eruption status. The most common attachment sites of the gubernacular canal to the dental follicle were on the occlusal aspect of the follicle and centrally in the buccal-lingual and mesiodistal directions. Therefore, the cases in which the attachment was located on the incisal/occlusal aspect of the follicle and centrally in the buccal-lingual and mesiodistal directions were classified as usual locations, while those at different sites were classified as unusual locations (Fig. 4) (6). In anterior/premolars, there were 15 cases with impacted teeth, of whom 12 cases had usual, and 3 cases had unusual attachment sites to the dental follicle. In molar teeth, there were 19 cases with impacted teeth, of whom 16 cases were related to the usual attachment site, and 3 cases were related to unusual attachment sites to the dental follicle.

## Discussion

In the present study, among 150 CBCT images assessed, the gubernacular canal was seen in 133 scans (88.7%). Nishida *et al*. (19) visualized the gubernacular canal for the first time on panoramic CBCT and multidetector CT scans. They reported that the gubernacular canal was present in 1.6% (one of 62) of central supernumerary teeth. In the study by Gaeta-Araujo *et al*. (6) on 598 CBCT samples, the authors detected gubernacular canal in 542 (90.6%) cases, which is consistent with the results found in the present study and the review study by Oda *et al*. (20).

The highest presence of the gubernacular canal was observed in teeth with normal eruption (92.1%), followed by impacted teeth (82.2%), and finally, teeth with delayed eruption (75%). In the study by Gaeta-Araujo *et al*. (6), the gubernacular canal was seen mostly in normal eruption (94.1%), followed by impacted teeth (87.1%) and delayed eruption (62.9%). The reason for the minor difference in these results can be related to the race and the quantity of population studied. We did not find a significant association between gubernacular canal presence and teeth eruption status, which is inconsistent with the study by Koc *et al*. (21), in which the authors concluded that the lack of gubernacular canals might suggest an abnormal tooth eruption pattern.

The mean length and diameter of the gubernacular canal in the molar teeth were larger than other teeth, which was also reported in the review study by Oda *et al*. (20). The mean gubernacular canal diameter in this study was 1.84 mm. The largest canal diameter was 3.40 mm. This value in the study by Nishida *et al*. (19) was seen between 1 and 3 mm. The reason for the discrepancy between these numbers can be the difference in the age and demographic conditions of the population studied.

In the anterior and premolars (teeth with predecessor primary tooth), the opening of the gubernacular canal in the alveolar crest was mostly on the lingual side. In molars, this structure was mostly located in the central part of the alveolar crest. These findings are consistent with our knowledge of the origin of the gubernacular canal in anterior and premolars, derived from deciduous teeth, and in molar teeth, originating from the posterior region of the dental lamina (6,22,23).

Unusual attachment sites of the gubernacular canal to dental follicles were mostly seen in abnormal eruptions. These findings are also in agreement with the study by Oda *et al*. (20). Therefore, in CBCT images, if the gubernacular canal is connected to an unusual location of the dental follicle, the probability of an abnormal eruption in this tooth will be higher (6,24). Overall, several factors can affect the process of tooth eruption, such as alveolar bone, dental follicles, osteoclasts, osteoblasts, and cytokines (6,23,25).

The clinical implications of this study are significant for dental practice. Dentists should be aware of the common occurrence of gubernacular canals (present in 88.7% of cases) and their potential influence on impacted teeth (27.3% of cases). Understanding the attachment sites (usual and unusual locations) is crucial for diagnosing complications accurately. Although variations in gubernacular canal length and diameter were observed, they provide valuable insights into developmental processes, aiding in treatment planning decisions. The study also emphasizes the essential role of CBCT in dental imaging, allowing precise visualization of these canals. Personalized dental care is key, necessitating individual assessments considering gubernacular canals’ presence and features. Timely dental evaluations are crucial, given the higher detection rate in normal tooth eruption cases, enabling early intervention and prevention of complications. Dentists must consider tooth type-specific variations in gubernacular canal characteristics, tailoring their approaches accordingly. A holistic approach, considering multiple factors, is vital in addressing cases of delayed or impacted tooth eruption. Lastly, lifelong dental vigilance, including regular check-ups and advanced imaging techniques, ensures comprehensive patient care across all age groups.

A limitation of the present study was its cross-sectional designation. So, it is proposed to design and carry out prospective longitudinal studies to discover the gubernacular canal changes during the tooth development and eruption.

## Conclusions

The findings of this study did not indicate a significant association between the presence of the gubernacular canal and the teeth eruption status. Moreover, there was no significant association between the opening of the gubernacular canal in the alveolar crest and eruption status. Finally, unusual attachment sites of the gubernacular canal to dental follicles were chiefly seen in abnormal eruption.
